# Correlates of work productivity and maternal competence after having a baby: the roles of mother-infant bonding and maternal subjective experiences

**DOI:** 10.1186/s12905-022-01958-w

**Published:** 2022-09-27

**Authors:** Pia Tohme, Rudy Abi-Habib

**Affiliations:** Department of Social and Education Sciences, School of Arts and Sciences, Lebanese American University, Beirut, Lebanon

**Keywords:** Maternal leave, Work productivity, Mother-infant bonding, Parental competence, Guilt, Stress

## Abstract

**Background:**

The law in Lebanon allows new mothers to take up to 10 weeks paid maternity leave, and some private organizations choose to give fathers 2 days of paternity leave in the absence of clear legislation. This falls short of the 6 months recommended for mothers in the literature.

**Methods:**

The sample consisted of 97 Lebanese mothers with children between 6 and 24 months of age. First, we examined correlations between the length of maternity leave and measures of work productivity and maternal sense of competence in their new role as a parent. Second, we explored correlations between the length of maternity leave and mother–child adjustment measures (mother-infant bonding, maternal stress, and parenting guilt). Finally, we looked for predictors of work productivity and maternal sense of competence in demographic variables, mother–child adjustment measures, and partner attachment scores.

**Results:**

Results showed significant correlations between work productivity and maternal sense of competence, irrespective of the length of maternity leave. Regression analyses showed that working for pleasure, mother-infant healthy bonding, and positive subjective experience of being back to work were significant predictors of work productivity, and that maternal stress predicted maternal competence.

**Conclusion:**

These findings are discussed within the framework of providing suggestions facilitating mothers’ transition back to work and increasing work productivity after having a baby.

## Introduction

Over the past few decades, economic and social demands have imposed longer working hours on parents, thus affecting the work-family balance [14]. This requires closer attention when it comes to new parents and the transition into parenthood, as these factors constantly and dynamically affect parental leave policies.

One of the reasons for which the optimal length of parental leave is crucial is its effect on decreasing parental mental health problems and stress, with mothers taking at least 12 weeks maternity leave showing less depressive symptoms than those who taking less than 12 weeks off [8, 12]. Scholars have found that maternal working hours post-delivery are positively correlated with depressive symptoms and parenting stress, as well as negatively associated with self-rated overall health, despite the fact that no significant correlation has been found with the quality of parenting when the child was 6 months old [9]. In other words, Chatterji et al. [9] found that going back to work too soon after delivery might have a negative effect on maternal mental health, and therefore potentially on work productivity, but not on the quality of parenting and sense of competence.

Other factors have been found to play a role in determining the quality of the early mother-infant bonding relationship. Indeed, whether or not employed, mothers tend to be responsible for most household and childcare responsibilities [11]. It is therefore argued that working mothers, especially those who do not have support in childcare, perceive themselves as having additional challenges in trying to fulfill their responsibilities both, at home and work. The inability to achieve these set goals and expectations has been shown to increase guilt and stress feelings in working mothers [5, 11] which, it can be argued, will in turn affect the quality of their bonding relationship with the child.

One cannot discuss paid maternity leave without including the effect it has on the length of breastfeeding and its relationship with mother-infant bonding, with mothers benefiting from longer paid maternity leave being more likely to exclusively breastfeed their child for longer [2]. This was shown to positively affect child development and maternal mental health as well as maternal role performance and sense of competence and efficacy [26]. Both the American Academy of Pediatrics [1] and the World Health Organization [28] have recommended that mothers should exclusively breastfeed until the child is at least 6 months old, suggesting that it promotes the child’s psychosocial development and mother-infant bonding as well as reduces the risks of early infection and diseases.

Fathers have rarely been included in studies investigating the impact of parental leave on early development and parental competence; however, they play an undeniable role as the stability and quality of the couple’s relationship have been found to have a crucial effect on adjustment to parenthood [14, 25]. One way to include fathers is to focus on the couple’s attachment relationship, as a recent study found that maternal romantic attachment was positively correlated with prenatal attachment to the baby. Furthermore, insecure romantic attachment was found to constitute a risk factor for lower postpartum mother-infant bonding [10].

### The Lebanese context

To our knowledge, studies investigating the effects of the length of maternity leave on breastfeeding practices and parental stress are scarce in Lebanon and the Arab region, with little to no advocacy in relation to the effects of maternity leave length on the mental health of both mother and child [13]. The increased role of women in the workplace in the Arab region, partly due to society’s gradual move toward more gender equality and away from a patriarchal society [15, 20] could also be seen as an important factor to take into account to better understand the relevance of this study. Considered one of the shortest in the Arab region and lagging behind international standards, Lebanese law only allows new mothers 70 days of paid maternity leave [13]. This could be discouraging some women to have children or to work, as they might find it difficult to balance between motherhood and a career especially with paternity leave being almost non-existent in the Arab region, implying it is the mother’s role to care for the child [15]. Saade et al. [22] have shown that 73% of Lebanese women are not satisfied with the length of paid maternity leave and that the premature return to work had negative consequences on maternal mental and physical health and led to shorter duration of breastfeeding. It is argued that new mothers are somewhat forced to “make a mental cost–benefit analysis, weighing the value of their income against the extreme difficulty of having full responsibility for an infant while trying to continue in an unsupportive employment environment” [15]. This often leads women to quit their jobs [22].

Our study is unique in that it is the first conducted in Lebanon that focuses not only on parental leave and parental mental health but also includes their effects on work productivity and parental self-rated competence in mothers, as well as on the perceived mother-infant bond, maternal guilt and stress. It also includes factors such as length of maternity leave, whether the mother was breastfeeding or not, getting help from other family members, working because of necessity or for pleasure, subjective experience of being back at work, which will be coined as demographic factors. This study therefore aims to (1) look at the correlation between length of maternity leave, work productivity, and maternal competence, (2) investigate the correlation between length of maternity leave, working because of necessity or for pleasure, subjective experience of being back at work, and measures of mother–child adjustment including mother-infant bonding, maternal stress, and parenting guilt, and (3) look for predictors of work productivity and maternal competence in demographic variables, as well as mother–child adjustment measures, and attachment between partners.

## Method

### Design

This study was a cross-sectional design, with data collected over a period of 2 months.

### Participants

We aimed to collect data from mothers and fathers and received 99 responses in total. However, given that only 2 male participants completed the survey, they were excluded from the analysis. Thus, our sample consisted of 97 mothers, all married, aged between 25 and 42 years (*M* = 32.52, *SD* = 3.61), with children (55 boys and 42 girls) aged between 6 and 24 months (*M* = 14.42, *SD* = 6.24). If a mother had more than one child within this age range, she was asked to rate statements about one of them only. The inclusion criteria included being a Lebanese parent having a child between 6 and 24 months, residing and working in Lebanon. There were no exclusion criteria.

### Measures

The *Health and Work Questionnaire* (HWQ) [24] is a 24-item self-report questionnaire relating one’s subjective assessment of workplace productivity and the worker’s health. Each item (for instance, “How personally rewarding did you find your work this week”) is rated on a 10-point Likert scale. A total HWQ score is calculated by computing the mean of item scores, after reverse coding all negative worded statements so that higher scores always indicate higher satisfaction. The HWQ total score showed high internal consistency of *α* = 0.81 [24], with *α* = 0.96 in our sample.

Two questions were added to the demographics sheet “Would you say you work mainly for financial necessity or pleasure?” and “Please rate your experience of being back to work after parental leave” in order to have a more subjective rating of participants’ working experience. Both questions were rated on a 0–10 Likert scale with 0 reflecting “working as a necessity” and “finding it difficult being back at work” respectively and 10 reflecting “working for pleasure” and”finding it easy being back at work” respectively.

The *Parenting Sense of Competence* (PSOC) [19] is a 17-item self-report scale scored on a 6-point Likert scale yielding a total score, as well as scores on two subscales measuring parental satisfaction (“Even though being a parent could be rewarding, I am frustrated now while my child is at his/her present age”) and parental efficacy (“Being a parent is manageable, and any problems are easily solved”) in relation to their child. Scores are computed by calculating the mean of answer scores, after reverse coding negatively worded items so that higher scores indicate higher parenting efficacy and satisfaction. The scale has been found to have good internal consistency with *α* = 0.79 for the total parental competence score, *α* = 0.75 for the parental satisfaction subscale, and *α* = 0.76 for the parental efficacy [19] and *α* = 0.82, *α* = 0.76, and *α* = 0.78 respectively in our sample.

The *Postpartum Bonding Questionnaire* (PBQ) [7] is a 25-item self-report questionnaire (including items such as “I resent my baby”) scored on a 5-point Likert sale. It reflects a parent’s feelings and attitudes towards their infant, with lower scores suggesting healthy positive bonding. In our sample, the PBQ has been found to have high internal consistency with *α* = 0.97.

The *Parental Stress Scale* (PSS) [3] is an 18-item self-report questionnaire (including items such as “I am happy in my role as a parent.”) focusing on feelings and perceptions about the experience of being a parent. A total score is computed by summing item scores, after reverse coding negatively worded statements so that higher scores reflect higher parenting stress. This scale has been found to have high internal consistency with *α* = 0.83 [3] and *α* = 0.85 in our sample.

The *Guilt about Parenting Scale* (GAPS) [17] is a brief 10-item questionnaire (including items such as “I often worry I am not as good a parent as I should be”) rated on a 7-point Likert scale. It provides an overall score of parenting guilt by summing item scores, with higher scores indicating higher levels of guilt. This scale has been found to have high internal consistency of *α* = 0.89 [17] and *α* = 0.85 in our sample.

The *Short Experiences in Close Relationships*-*Revised* (ECR-R short) [27] is a 12-item self-report questionnaire scored on a 7-point Likert scale. Two subscale scores assessing attachment avoidance (including 6 items such as “I try to avoid getting too close to my partner”) and attachment anxiety (including 6 items such as “I need a lot of reassurance that I am loved by my partner”) in romantic relationships can be calculated by computing the mean of scores. Higher scores indicate higher avoidance and anxiety (insecure attachment). The validity of the short version was found to be equivalent to that of the original scale [16] with *α* ranging between 0.77 and 0.86 for attachment anxiety and 0.78 and 0.88 for attachment avoidance [27]. In our sample we found *α* = 0.72 and *α* = 0.79 for the attachment anxiety and attachment avoidance subscales respectively.

The survey also included a demographics sheet including information with regards to participants’ age and gender, the child’s age and gender, marital status, employment status (working or not), availability of caretaking, length of maternity leave, and whether the mother breasted or not.

### Procedure

After receiving ethical approval from the Lebanese American University Institutional Review Board, we proceeded to collecting data using an online survey disseminated to acquaintances and through social media platforms. All questionnaires were used in their original English form. Filling out the questionnaires took approximately 15 min. All data was automatically saved anonymously through the Blue online platform.

### Statistical analysis

Data was analyzed using SPSS. This first aim of this study was to look at the correlations between length of maternity leave, work productivity (HWQ), and maternal competence (PSOC Total, PSOC Satisfaction, PSOC Efficacy). We used Pearson correlations for this purpose. Second, we investigated the correlation between length of maternity leave, working because of necessity or for pleasure, subjective experience of being back at work, and measures of mother–child adjustment, including mother-infant bonding (PBQ), maternal stress (PSS), and parenting guilt (GAPS) using Pearson correlations. Third, we ran two multiple linear regression models, with work productivity (HWQ Total) as the dependent variable in the first and maternal competence (PSOC Total) as the dependent variable in the second. Predictors in both models included measures of mother–child adjustment (PBQ, PSS and GAPS), demographics factors (length of maternity leave, whether the mother was breastfeeding or not, getting help from other family members, working because of necessity or for pleasure, subjective experience of being back at work), as well as attachment to partner (ECR-R).

## Results

The sample consisted of 97 mothers. Almost all (97%) women were employed, and 59% reported receiving help with child care. Participants reported taking between 7 and 365 days for maternity leave (*M* = 75.70. *SD* = 40.81), but 91% of mothers reported wanting to have had longer maternity leave. Most women (86%) breastfed their child. Means and standard deviations of the main variables are presented in Table 1.

**Table 1:** Descriptive statistics of main variables

	*M*	*SD*
Mother’s age	32.52	3.61
Child’s age (in months)	14.42	6.24
Parental leave length (in days)	75.70	40.81
Working for necessity/pleasure	4.67	2.88
Experience back at work	3.56	2.72
HWQ	5.86	1.17
PSOC total	3.99	0.69
PSOC satisfaction	3.79	0.87
PSOC efficacy	4.08	0.81
PBQ total	27.40	29.61
PSS total	41.28	9.63
GAPS total	48.25	10.85
ECR-R anxiety	3.61	1.19
ECR-R avoidance	2.44	1.09

Working for necessity/pleasure = “Would you say you work mainly for financial necessity or pleasure?” with 0 = “working as a necessity” and 10 = “working for pleasure”, Experience back at work = “Please rate your experience of being back to work after parental leave”. with 0 = “finding it difficult being back at work” and 10 = ”finding it easy being back at work”, HWQ Total = work productivity total score, PSOC Total = parental sense of competence total score, PSOC Satisfaction = parental sense of competence (satisfaction subscale), PSOC Efficacy = parental sense of competence (efficacy subscale), PBQ Total = parent–child bonding total score, PSS Total = parental stress total score, GAPS Total = parental guilt about parenting total score, ECR-R anxiety = attachment anxiety towards partner score, ECR-R avoidance = attachment avoidance towards partner score

Correlational analyses between length of maternity leave, work productivity and maternal competence were not significant except between work productivity and maternal efficacy, with *r* (95) = 0.24, *p* = 0.20 (Table 2).

**Table 2:** Correlations between Length of Maternity Leave, Work Productivity and Maternal Competence

	1	2	3	4	5
1 Parental leave length in days	1	0.08	0.03	− 0.02	0.04
2 HWQ total		1	0.17	0.02	0.24^*^
3 PSOC total			1	0.79^**^	0.88^**^
4 PSOC satisfaction				1	0.41^**^
5 PSOC efficacy					1

HWQ Total = work productivity total score, PSOC Total = parental sense of competence total score, PSOC Satisfaction = parental sense of competence (satisfaction subscale), PSOC Efficacy = parental sense of competence (efficacy subscale)

*p < 0.05, **p < 0.01

Correlational analyses between the length of maternity leave and measures of mother–child adjustment were not significant except between maternal stress and guilt, with *r* (95) = 0.22, *p* = 0.03 (Table 3).

**Table 3:** Correlations between length of maternity leave and maternal bonding, stress and guilt

	1	2	3	4
1 Parental leave length in days	1	− 0.18	− 0.11	− 0.13
2 PBQ total		1	0.11	0.10
3 PSS total			1	0.22*
4 GAPS total				1

PBQ Total = parent–child bonding total score, PSS Total = parental stress total score, GAPS Total = parental guilt about parenting total score

*p < 0.05, **p < 0.01

Significant correlations were found between the experience of being back at work and maternal guilt, *r* (95) = − 0.19, *p* = 0.05, work productivity and bonding, *r* (95) = 0.23, *p* = 0.02, maternal competence and stress *r* (95) =  − 0.63, *p* = 0.001, and guilt *r* (95) =  − 0.27, *p* = 0.01, as well as between maternal stress and guilt, *r* (95) = 0.22, *p* = 0.03 (Table 4).

**Table 4:** Correlations between maternal ratings of their experience back at work, work productivity, maternal competence and measures of parenting

	1	2	3	4	5	6
1 Experience of being back at work	1	− 0.08	0.18	− 0.01	− 0.07	− 0.19*
2 HWQ Total		1	0.17	0.23*	− 0.12	− 0.19
3 PSOC Total			1	− 0.05	− 0.63**	− 0.27**
4 PBQ Total				1	0.11	0.10
5 PSS Total					1	0.22*
6 GAPS Total						1

HWQ Total = work productivity total score, PSOC Total = parental competence total score, PBQ Total = parent–child bonding total score, PSS Total = parental stress total score, GAPS Total = parental guilt about parenting total score

*p < 0.05, **p < 0.01

Similarly, significant correlations were found with work productivity, *r* (95) = 0.32, *p* = 0.001, maternal competence, *r* (95) = 0.32, *p* = 0.001, maternal stress, *r* (95) = − 0.22, *p* = 0.03, and maternal guilt, *r* (95) = − 0.24, *p* = 0.02.

Results of the first regression indicated that three predictors explained 24.9% of the variance in work productivity (*R*^*2*^ = 0.25, *F* (10, 86) = 2.86, *p* = 0.004). It was found that working for pleasure significantly predicted work productivity (*β* = 0.33, *p* = 0.002), as did ratings of the experience of being back at work (*β* = − 0.21, *p* = 0.04), and mother-infant bonding (*β* = 0.23, *p* = 0.02; Table 4). The second regression was also significant, indicating that one predictor, maternal stress (*β* = − 0.54, *p* = 0.001), significantly explained 47.8% of the variance in maternal competence (*R*^*2*^ = 0.48, *F* (10, 86) = 7.88, *p* = 0.001; Table 5).

**Table 5:** Regression models looking for predictors of work productivity and parental competence

	Work productivity			Parental competence		
	*B*	*SE B*	*β*	*B*	*SE B*	*β*
Length of parental leave	0.21	0.46	0.05	− 0.01	0.01	− 0.09
Breastfeeding (yes/no)	31.46	51.85	0.06	0.10	0.16	0.05
Getting help from other family members	− 5.97	37.92	− 0.02	− 0.06	0.12	− 0.04
Work as necessity or pleasure	21.71	6.76	0.33**	0.04	0.02	0.16
Experience of being back at work	− 14.45	6.93	− 0.21*	0.02	0.02	0.09
PBQ total	1.48	0.63	0.23*	0.00	0.01	− 0.01
PSS total	− 0.31	2.02	− 0.02	− 0.04	0.01	− 0.54***
GAPS total	− 2.73	1.75	− 0.16	− 0.01	0.01	− 0.09
ECR-R anxiety	10.45	17.38	0.07	− 0.01	0.05	− 0.012
ECR-R avoidance	− 31.69	18.72	− 0.18	− 0.09	0.06	− 0.14
*R*^*2*^	0.25			0.48		
*F*	2.86**			7.88***		

B = Non-standardized coefficients, SE B, = Standard error of non-standardized coefficients, β = standardized coefficients, PBQ Total = parent–child bonding total score, PSS Total = parental stress total score, GAPS Total = parental guilt about parenting total score, ECR-R anxiety = attachment anxiety towards partner score, ECR-R avoidance = attachment avoidance towards partner score

*p < 0.05, **p < 0.01, ***p < 0.001

## Discussion

This study is unique in that it focused on the relationships between maternity leave length, maternal sense of competence, work productivity and markers of mother-infant bonding in a sample of Lebanese mothers with children between 6 and 24 months. First, we found a small but significant correlation between work productivity and mothers’ sense of competence, irrespective of the length of maternity leave. This suggests that women’s own perceptions of their roles as caregivers and as mothers play a more crucial role than the amount of time spent with their child as part of maternity leave in explaining work productivity after having a baby. This is in line with Chatterji et al. [4] who highlighted that shorter maternity leave has a detrimental effect on maternal mental health but not on mothers’ sense of competence and parenting quality. Similarly, there was a significant correlation between work productivity and healthy mother–child bonding which emphasizes the importance of internal factors (perceived sense of competence and subjective ratings of healthy parent-infant relationship) in understanding levels of work productivity after having a baby. In other words, a woman’s perception of her sense of self as a mother plays a more important role in increasing her sense of self as a professional than the length of maternity leave.

Second, we found a significant correlation, albeit small, between maternal guilt and stress. This echoes the literature suggesting difficulties some working mothers might face in balancing their two roles and increased responsibilities, thus leading to increased stress and feelings of guilt in their parenting [5, 11, 15, 20]. Noteworthy are the non-significant correlations between maternity leave length and other key variables in this study, especially that 91% of mothers in this sample wished they had longer maternity leaves, in line with Saade et al. [22] results in a Lebanese sample. This was surprising given that 49% of mothers had taken longer than the government-allowed 70 days paid maternity leave and the fact that the length of maternity leave was not significantly correlated with stress, guilt, parental competence or work productivity.

In attempting to further elucidate this finding, we explored the correlation between key variables and mothers’ subjective ratings of their experience of being back at work. We found that the more negative they rated this experience, the higher the guilt. In addition, the more mothers reported they were working due to financial necessity rather than pleasure, the higher the stress and guilt and the lower the sense of maternal competence and work productivity. This sheds light on the importance of internal factors in determining a mother’s parental competence and work productivity after having a baby. More specifically, a mother’s experience of being unhappy or forced to leave her child at home appears to be the most significant factor affecting her experience of herself as a working parent and her feelings of guilt. These findings are in line with previous studies in the Arab region describing the mental cost–benefit analysis between the two roles, motherhood versus career, leading some women to have to choose only one [15, 22]. This is an important finding promoting the advocacy of new laws facilitating a woman’s transition into motherhood without impacting her role or position in the workplace.

Furthermore, working for pleasure rather than for financial necessity, perceived mother-infant healthy bonding and positive subjective experience of being back to work were found to be significant predictors of work productivity. This reflects the idea that a supportive workplace environment and a woman’s sense of flexibility in her ability to choose whether or not to work is an important predictor of her work productivity. This echoes El Awady’s [15] argument of the mental cost–benefit analysis of whether and when to go back to work. Future research could explore factors that promote mothers’ positive subjective experience at work after having a baby, such as perceived workplace support and flexibility [18, 21] or the availability of a private space to pump breastmilk at work [6]. It would also be of interest to explore the role of partner support, rather than only the quality of the romantic attachment relationship, in women’s subjective experience of being back at work. It could be hypothesized that the more support a woman gets from her partner, whether in taking care of the child or encouraging her to find a balance between her role as a professional and a mother, could play a crucial role in her subjective experience of being back a work and of being a mother.

Finally, maternal self-rated stress was found to be the only significant predictor of mothers’ sense of competence, explaining 70% of the variance. Indeed, in line with previous research, the more stress a mother perceived in her role as a parent, the less likely she was to rate herself high in terms of parental competence, including both, parental self-efficacy and satisfaction [5, 11]. It should be noted that, contrary to our expectations, getting help with caring for the child, partner support, breastfeeding and length of maternity leave were not significant in either model. This could be explained by the fact that, in previous studies, maternal mental health markers such as depression or anxiety, played a role in the significant correlation between these variables [2, 4, 22, 23, 26]. Maternal mental health was not explored in this study and future research could look at the mediating role of maternal mental health in explaining the relationship between these variables in Lebanese mothers.

Despite the uniqueness of this study, it is not without its limitations. First, this study is cross-sectional with a small sample size, which could limit the generalizability of results to the Lebanese population as a whole. Due to the COVID-19 pandemic, the survey was only sent in English to participants having access to the internet and only those in specific regions of the country were able to access it, potentially leading to selection bias. Also, the vast majority of our participants responded to the survey from home due to the COVID-19 lockdown, which might have had an effect on their perception of the constructs at hand. Second, all measures were self-reported questionnaires and it was difficult to differentiate between different types of stressors, especially given the economic hardship and COVID-19 lockdowns which could have skewed the results in relation to maternal stress. This could have entailed a recall bias whereas participants fail to remember some events or their effects. Third, while we sought to investigate both parents, only mothers responded to our questionnaires. Out of the 99 responses, we only had two men, while we advertised the study for both parents. This can be explained by the fact that in Lebanon, a patriarchal society, fathers do not see themselves involved in the immediate life of their child after birth. However, this point should be further explored given the rapidly-changing gender roles expectations due to Western influences; while some still adopt a rather traditional father role, others have assumed a more involved attitude, dividing the child-related chores with their wives. This said, it can be argued that these fathers will be negatively impacted by the inexistent paternity leave (there is as yet no official paternity leave in Lebanon), which might not only be negative on the fathers’ physical and mental health but also possibly affect their capacity to support their wives, hence weakening the couples’ structure as a whole. Lastly, it is important to note that the significant correlations between maternal stress, maternal guilt, and work productivity became insignificant when entered in the regression model, but that mother-infant bonding was a significant predictor of work productivity. It would therefore be interesting for future studies to explore the mediating roles of maternal guilt and stress in the relationship between healthy mother-infant bonding and higher work productivity, in an attempt to devise support groups empowering women’s sense of identity as mothers and professionals.

## Conclusion

In conclusion, despite the small correlations found in this study, we could build on these preliminary findings to suggest directions to facilitate mothers’ transition back to work and increase work productivity after having a baby. First, support groups can be created for mothers targeting stress and guilt management, providing them with strategies to balance their roles as mothers and workers. Multidisciplinary practices are increasing in Lebanon and these groups could be run through a collaboration between health care professionals (such as pediatricians or gynecologists) and psychologists. Second, focus group discussions can be conducted to better understand mothers’ perceptions of a positive work environment, as this factor was found to be crucial in decreasing maternal guilt and increasing work productivity. Finally, it takes two to tango; even though pregnancy and breastfeeding are specific to mothers, the role of fathers should not be reduced to maternal support solely. Healthy father-child bonding could well be critical to the child’s physical and mental health and should be further investigated in this specific part of the world considered simultaneously collectivistic and individualistic.

## Data Availability

All data and materials are available upon reasonable request from the corresponding author.
